# Regulatory non-coding RNAs: a new frontier in regulation of plant biology

**DOI:** 10.1007/s10142-021-00787-8

**Published:** 2021-05-20

**Authors:** Sailaja Bhogireddy, Satendra K. Mangrauthia, Rakesh Kumar, Arun K. Pandey, Sadhana Singh, Ankit Jain, Hikmet Budak, Rajeev K. Varshney, Himabindu Kudapa

**Affiliations:** 1 Center of Excellence in Genomics & Systems Biology (CEGSB), International Crops Research Institute for the Semi-Arid Tropics (ICRISAT), Hyderabad, India; 2 Crop Improvement Section, ICAR-Indian Institute of Rice Research, Hyderabad, India; 3 Department of Life Sciences, Central University of Karnataka, Karnataka, India; 4 College of Life Sciences, China Jiliang University, Hangzhou, China; 5 Montana BioAgriculture, Inc., Missoula, MT USA; 6 State Agricultural Biotechnology Centre, Centre for Crop and Food Innovation, Murdoch University, Murdoch, Western Australia Australia

**Keywords:** Regulatory non-coding RNAs, Biogenesis, Degradation, IsomiRs, Stress response, Crop improvement

## Abstract

Beyond the most crucial roles of RNA molecules as a messenger, ribosomal, and transfer RNAs, the regulatory role of many non-coding RNAs (ncRNAs) in plant biology has been recognized. ncRNAs act as riboregulators by recognizing specific nucleic acid targets through homologous sequence interactions to regulate plant growth, development, and stress responses. Regulatory ncRNAs, ranging from small to long ncRNAs (lncRNAs), exert their control over a vast array of biological processes. Based on the mode of biogenesis and their function, ncRNAs evolved into different forms that include microRNAs (miRNAs), small interfering RNAs (siRNAs), miRNA variants (isomiRs), lncRNAs, circular RNAs (circRNAs), and derived ncRNAs. This article explains the different classes of ncRNAs and their role in plant development and stress responses. Furthermore, the applications of regulatory ncRNAs in crop improvement, targeting agriculturally important traits, have been discussed.

**Supplementary Information:**

The online version contains supplementary material available at 10.1007/s10142-021-00787-8.

## Introduction

**Fig. 1:**
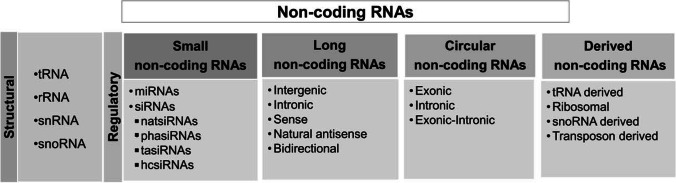
Classification of non-coding RNAs: different classes and subclasses of structural and regulatory non-coding RNAs

Crop plants adapt to different regulatory mechanisms to accomplish sustainable productivity. A myriad of non-coding RNAs (ncRNAs) are important players in these regulatory networks. In the recent past, research on ncRNAs has been accelerated with the advent of deep sequencing technologies in the field of molecular biology. The ncRNAs derived from transcriptionally active genes do not encode a functional protein (Palazzo and Lee 2015). The structural class of ncRNAs comprises ribosomal RNA (rRNA), transfer RNA (tRNA), small nuclear RNA (snRNA), and small nucleolar RNA (snoRNA) (Fig. 1). The regulatory ncRNAs (rncRNAs) are broadly classified into long ncRNAs (lncRNAs, > 200 nt) and small ncRNAs (sncRNAs, 18–30 nt). Furthermore, several studies have reported the participation of other regulatory ncRNAs such as “circular” RNAs (circRNAs) and derived ncRNAs in plant processes (Sablok et al. 2016; Zhu et al. 2018).

The first ncRNA characterized in baker’s yeast was alanine tRNA (Holley et al. 1965). The catalytic role of the RNA in the 1980s opened a new perspective for researchers to understand the complex role of different ncRNAs (Morris and Mattick 2014). Subsequently, discovery of the regulatory action of ncRNAs in *Caenorhabditis elegans* was emerged as a big revolution in the world of “nc” RNA and led to the identification of different classes of ncRNAs in humans, animals, and plants (Chen 2009; Lee et al. 1993; Mattick and Makunin 2006; Yu et al. 2019). Besides, technological advancements also paved the way for identification of several other regulatory ncRNAs modulating the expression of protein-coding genes in various cellular processes by interacting with different molecular pathways. However, the gathered knowledge of ncRNAs in plants is less compared to that in animals. In this review, we present the updates on the diverse regulatory role of ncRNAs in plant biology including ncRNA variants/isoforms, circRNAs, and derived ncRNAs. Furthermore, examples of ncRNA-mediated regulation in the development of plant phenotypes with improved agronomic traits and the possible ways to utilize this information in crop improvement programs are discussed.

## Biogenesis

**Fig. 2:**
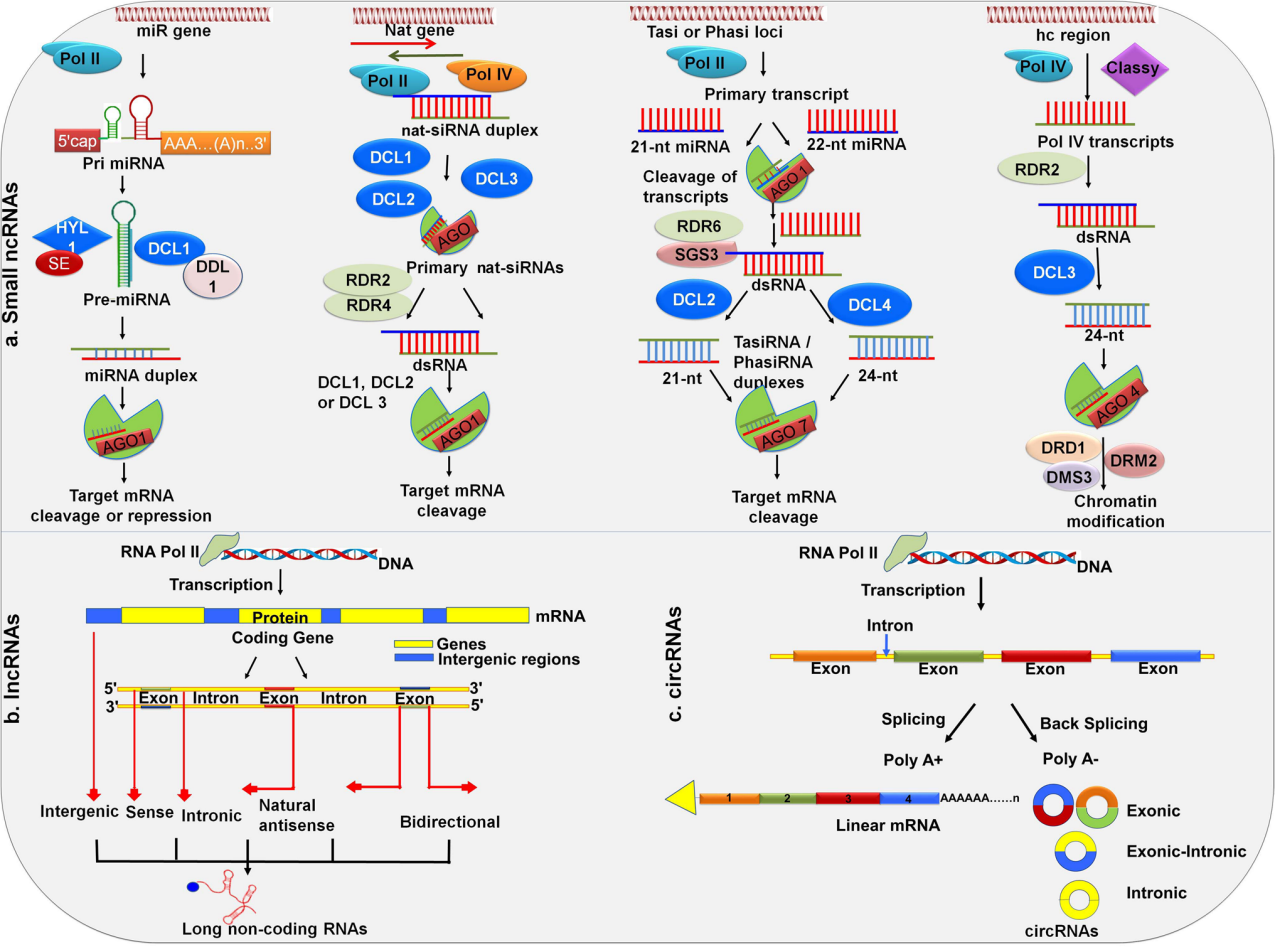
Canonical biogenesis of regulatory non-coding RNAs. **a** Small ncRNA biogenesis (left to right): microRNAs (miRNAs) transcribed from respective miR genes through the intermediate stem-loop structures called precursor miRNAs (pre-miRNAs) and miRNA duplexes by polymerase II (pol II) and other proteins. Further mature miRNA strand is incorporated to Argonaute (AGO) I for post-transcriptional gene silencing (PTGS) by target cleavage or repression. Next to the miRNAs, a class of natural antisense RNAs (natsiRNAs or nat-siRNAs) derived from the *nat* genes by the action of pol II or pol IV by forming double-stranded RNAs (dsRNAs) as intermediates either from overlapping loci or from complementary loci to generate *cis* and *trans* natsiRNAs. *Trans* acting siRNAs (tasiRNAs or ta-siRNAs) or phasiRNAs are transcribed from respective *tasi* or *phasi* genes by pol II through the formation of dsRNAs as intermediates. Action of RNA-dependent RNA polymerase 6 (RDR6) on dsRNAs results in the formation of 22-nt or 24-nt tasiRNAs or phasiRNAs. natsiRNAs/tasiRNAs/phasiRNAs are involved in target gene cleavage. Heterochromatic siRNAs (hcsiRNAs) are derived by the transcription of heterochromatin regions with Pol IV and RDR2 through the formation of intermediate dsRNAs. Further subsequent process of dsRNAs results in the formation of 24-nt hcsiRNAs that mainly involves chromatin modifications. **b** Long non-coding RNAs (lncRNAs): LncRNAs are transcribed by Pol II, and based on their relative position of their transcription from the genome, lncRNAs are classified in to intergenic, sense, intronic, natural antisense, and bidirectional. **c** Circular RNAs (circRNAs): circRNAs are derived from the exons, introns, or exonic-intronic regions through back-splicing of protein-coding genes

**Fig. 3:**
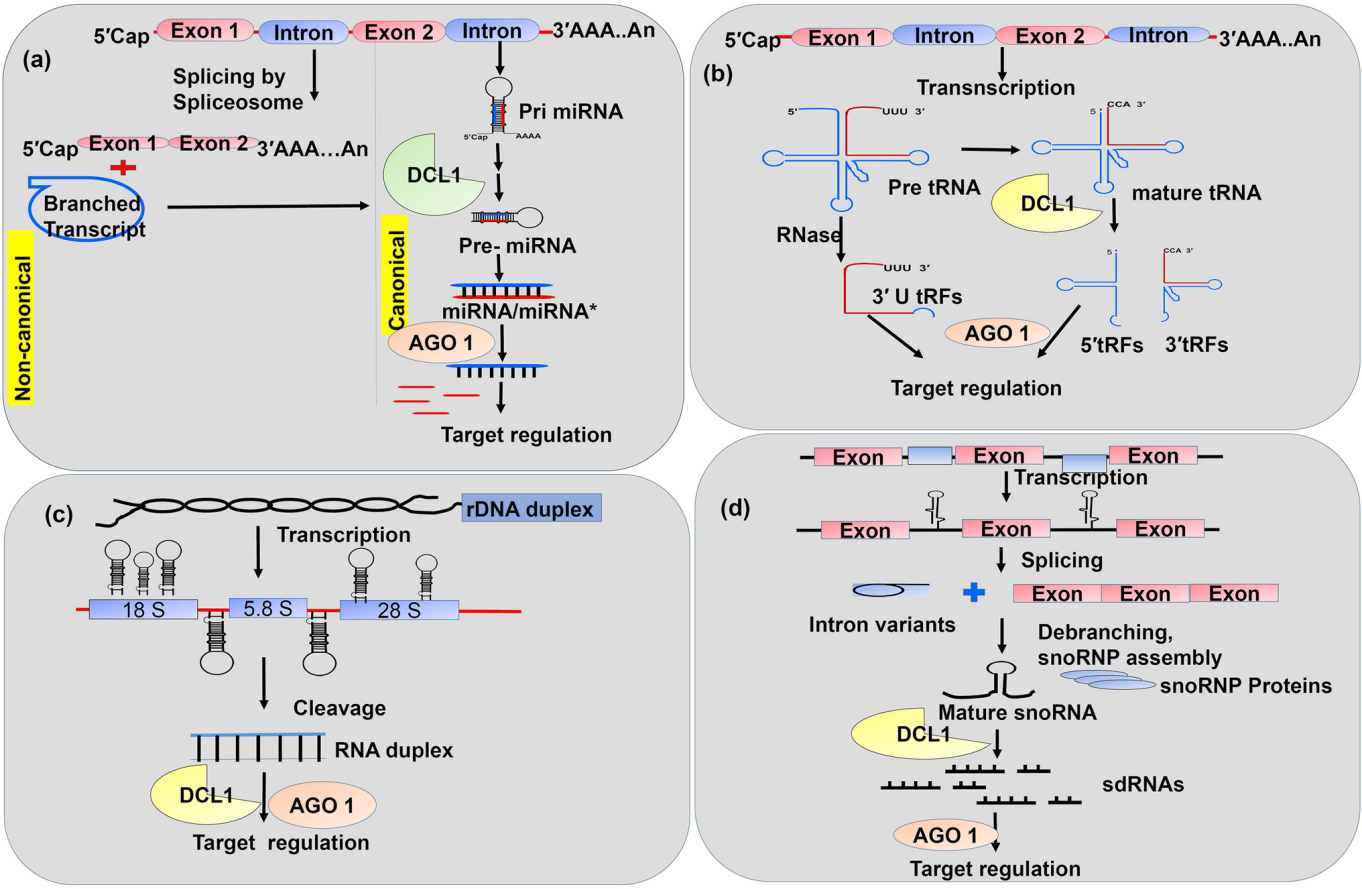
Non-canonical biogenesis of non-coding RNAs. **a** Non-canonical biogenesis of miRNAs occurs through mirtron-like transcripts which can be spliced and debranched in to pre-miRNA hairpins that bypasses the pri-miRNA step in canonical biogenesis. Debranched mirtron-like transcripts enters canonical miRNA pathway and later cleaved by Dicer like1 (DCL1) and Argonaute 1 (AGO1) proteins. **b** tRNA-derived non-coding RNAs pathway includes the action of RNases on both precursor tRNAs (pre tRNA) and mature tRNAs that give rise to small RNAs like 3′ U tRFs, 5′ tRFs, and 3′ tRFs. **c** Small RNA duplexes derived from ribosomal DNA (rDNA) through transcription and subsequent cleavage by DCL1 functions like miRNAs by entering in to AGO pathway. **d** Small RNAs derived from snoRNAs (sdRNAs) regulate their target transcripts by entering in to DCL1 and AGO1 pathway like miRNAs

ncRNA biogenesis is a complex phenomenon and can be derived from two major pathways: canonical and non-canonical. Canonical pathway denotes the ncRNA synthesis by classical steps (Fig. 2), while non-canonical pertains to non-classic/non-regular ways that follow alternative pathways (Fig. 3).

### Canonical biogenesis

#### miRNAs

Canonical biogenesis of miRNAs and other siRNAs have been well-described earlier (Borges and Martienssen 2015). In brief, the miRNA biogenesis includes the synthesis of long primary transcripts called primary miRNAs (pri-miRNAs) with the aid of RNA Polymerase (Pol) II (Pol II), and it is further processed into precursor miRNAs (pre-miRNAs/premiR) by a set of proteins — DICER-LIKE 1 (DCL1), HYPONASTIC LEAVES 1 (HYL1), and SERRATE (SE) (Rogers and Chen 2013). Subsequent processing of pre-miRNA to miRNA/miRNA* duplex followed by the transfer of duplex from the nucleus to cytoplasm, where the preferential loading of mature miRNA to RNA-INDUCED SILENCING COMPLEX (RISC) occurs and reaches its target mRNA transcript by binding with ARGONAUTE 1 (AGO 1) protein. Thus, the regulation of target gene expression occurs either by transcript cleavage or by translational repression, and the miRNA* strand gets degraded (Borges and Martienssen 2015; Budak et al. 2015). miRNAs with 21-nt length are processed by DCL1 or DCL4 proteins, while 22 and 24-nt length miRNAs are processed by DCL2 and DCL3 proteins (Fig. 2a).

#### siRNAs

In contrast to miRNAs, siRNAs are either exogenous or endogenous, derived from the complementary long double-stranded RNAs by RNA-DEPENDENT RNA POLYMERASE (RDR) and cleaved into siRNAs by DCL1 proteins. Different classes of endogenous siRNAs in plants include secondary siRNAs (natural antisense transcript-derived siRNAs (natsiRNAs or nat-siRNAs), phased siRNAs (phasiRNAs), and *trans*-acting siRNAs (tasiRNAs or ta-siRNAs)) and heterochromatic siRNAs (hcsiRNAs) (Borges and Martienssen 2015) (Fig. 2a). natsiRNAs are the pairs of perfect complementary transcripts transcribed from endogenous coding or ncRNAs with the aid of Pol II/Pol IV, RDR2, and DCL1/DCL3-dependent pathway involved in post-transcriptional gene regulation through RNA interference (RNAi) (Borges and Martienssen 2015). tasiRNAs and phasiRNAs are generated from cleavage fragments of miRNA-target transcripts, and their precursors are transcribed from non-coding loci and protein-encoding genes, respectively, by Pol II. The subsequent cleavage of transcripts by miRNA-mediated AGO1/AGO7 results in single-strand RNAs (ssRNAs) and further into double-stranded RNAs (dsRNAs) by RDR6 and SUPPRESSOR OF GENE SILENCING3 (SGS3). Furthermore, conversion of dsRNAs into 21 or 24-nt siRNAs by DCL2 or DCL4 and loading into AGO1/AGO4 results in target cleavage (Allen et al. 2004). Importantly, phasiRNAs are triggered by either “one-hit” (one binding site in the target) or “two-hit” (two binding sites in the target) models (Fei et al. 2013). Contrarily, hcsiRNAs (23–24 nt) involve in the transcriptional gene silencing (TGS) by guiding the methylation of DNA and/or histones through the RNA-directed DNA methylation (RdDM) (Matzke et al. 2015) (Fig. 2a). These are derived from the transposable elements (TE) and repeats of hc regions by the action of Pol IV and CLASSY1 (CLSY1) followed by RDR2 and DCL3 to generate hcsiRNAs, that aid in the site-specific chromatin modifications (Yu et al. 2019).

#### lncRNAs and circRNAs

lncRNAs are usually derived from genomic regions that lack coding potential and possess the transcript length > 200 nt. The majority of lncRNAs that are transcribed by Pol II possess a 5′ cap and non-adenylated or poly-adenylated 3′ tail. Other RNA polymerases like Pol IV and Pol V also play a central role in the biogenesis of lncRNAs (Wierzbicki et al. 2008, 2009). lncRNAs transcribed from Pol IV and Pol V lack poly-A tails and merely less expressed when compared to lncRNAs derived from Pol II and play a significant role in driving RdDM (Budak et al. 2020; Zhou and Law 2015). Based on the biogenesis loci, lncRNAs are further classified as intergenic, intronic, sense or overlapping, antisense, and bidirectional (Budak et al. 2020) (Fig. 2b). lncRNAs regulate gene expression at transcriptional and post-transcriptional levels through different mechanisms. They act as scaffolds by interacting with chromatin regulatory proteins, as miRNA decoys (sponges/target mimics), and as mediators in epigenetic silencing (Wang et al. 2018a).

In addition, circRNAs, a distinct class of endogenous ncRNAs characterized by covalently closed structures without 5′ or 3′ ends, are derived through non-sequential back-end splicing from the precursor mRNAs by Pol II (Zhang et al. 2016a). These are categorized into exonic, intronic, intergenic, and exon-intronic, based on the derived genomic region and regulates the gene expression by acting as sponges for miRNAs (Sablok et al. 2016) (Fig. 2c).

### Non-canonical biogenesis

**Fig. 4:**
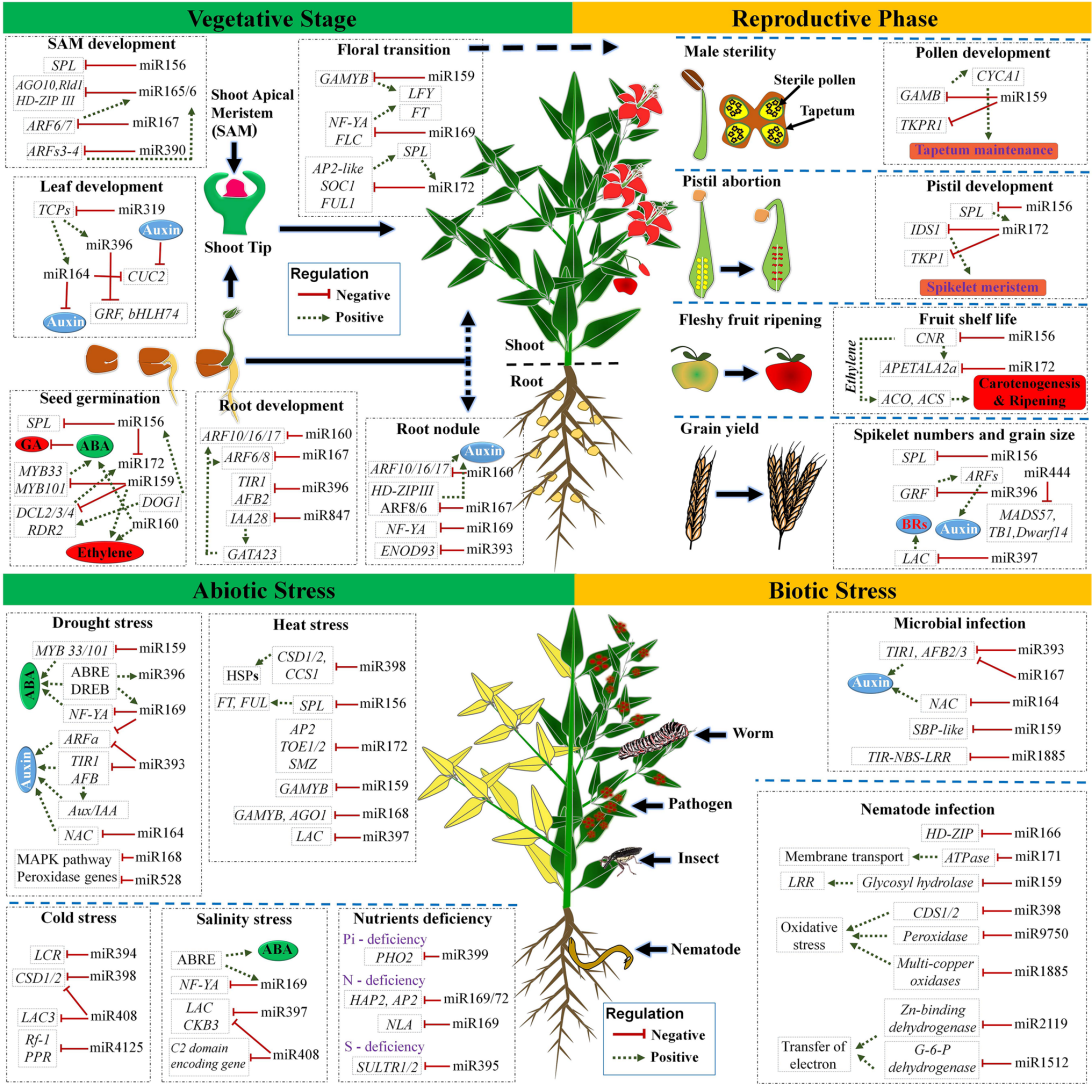
Regulation of miRNAs in plant development and stress mechanisms. Different miRNA-target modules involved in plant growth, development, abiotic and biotic stress episodes, and their regulation

**Fig. 5:**
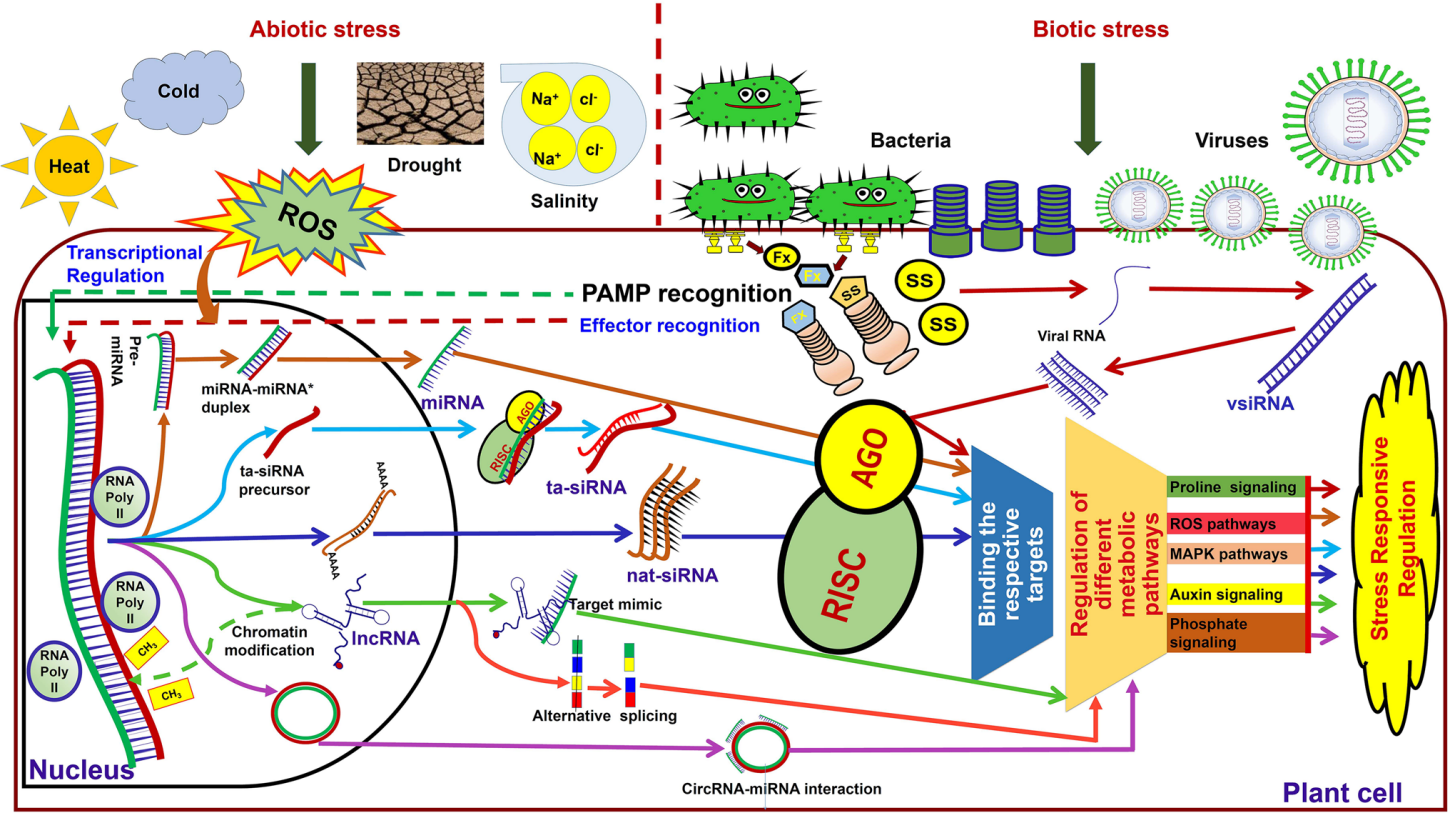
General model of stress-responsive regulation by regulatory non-coding RNAs in abiotic and biotic stresses. Abiotic and biotic stresses elicit the production of reactive oxygen species (ROS) and pattern-associated molecular pattern (PAMP) recognition in plants through signal perception. Synthesis of different classes of non-coding RNAs (ncRNAs) in response to ROS and PAMP is one of the defensive mechanisms in plants. miRNAs and other small ncRNAs thus produced in response to stress, bind to their respective target genes with the aid of Argonaute (AGO) proteins by forming RNA-induced silencing complex (RISC), and thus regulate the expression of target genes. Long non-coding RNAs (lncRNAs) regulate the gene expression by mimicking the miRNA targets or through alternative splicing or by chromatin modification. Circular RNAs (circRNAs) regulate the gene expression by acting as miRNA sponges. Successively, thus generated ncRNAs regulate the different metabolic pathways that primes in the stress-responsive regulatory mechanism. BE- bacterial effector, SS- silencing suppressor, dsRNA- double-stranded RNA, PRRs- pathogen recognition receptors, R proteins- resistance protein, RISC- RNA-induced silencing complex

Besides canonical, ncRNAs choose alternative routes for biogenesis through dicer-independent mechanisms and were described as “non-canonical” pathways, which include few miRNAs, and derived ncRNAs. Here, (1) pre-miRNA hairpin structures called “mirtrons” are generated through splicing mechanism instead of DCL1 (Budak and Akpinar 2015; Meng and Shao 2012), 2) DLC2, DCL3, and DCL4, act on long inverted repeat transcripts which results in miRNA species varying in length, (3) processing of pri-miRNAs in reverse orientation from loop to the base generates multiple duplexes of miRNA/miRNA* rather than a single duplex (Fig. 3a) (Budak et al. 2015; Sobkowiak et al. 2012). Sometimes, the unusual genetic loci can also harbor non-coding small RNAs. For example, (1) the derivatives of rRNAs, tRNAs, snoRNAs, snRNAs, and transposons are derived ncRNAs (Son et al. 2013), (2) small RNAs (18–26 nt) derived from tRNA are tRNA-derived fragments (tRFs), processed by either DICER or by Dicer-independent pathway through the action of ribonuclease (RNase) (Fig. 3b). Based on their derived region, the tRFs are categorized into 5′-tRFs, 3′-tRFs, and 3′-U tRFs. In plants, the regulatory role of tRFs through RNA degradation and translational inhibition was studied in Arabidopsis (Zhang et al. 2009; Zhu et al. 2018), (3) miRNAs derived from ribosomal DNA (rDNA) have also been reported in humans and plants (De Paola et al. 2016; Mangrauthia et al. 2018) (Fig. 3c), (4) small RNAs derived from snoRNA (sdRNA) possess a function as like miRNAs (Taft et al. 2009) (Fig. 3d), (5) TE-derived ncRNAs are transcribed from TE genomic regions through Pol II and processed by RDR6 and DCL2/4 to form 21 or 22-nt siRNAs and thus targets TE mRNAs for degradation in association with AGO1 (Cho and Paszkowski 2017). The activation of several TEs during hypomethylation results in epigenetically activated siRNAs (easiRNAs) (Creasey et al. 2014). Furthermore, TEs can establish RNA hairpin structures and proces them through miRNA biogenesis pathways to form TE-derived miRNAs (Creasey et al. 2014; Nosaka et al. 2012). In addition, TE-derived lncRNAs have also been reported in plants (Wang et al. 2015a).

The non-canonical pathways and unconventional genetic loci of ncRNAs biosynthesis in plants indicate the unknown complexity of gene regulation. More in-depth studies in this area of research will help in understanding the precise regulation of ncRNAs.

## ncRNAs isoforms, mechanisms, and biological significance

The combination of high-throughput sequencing technologies and bioinformatics advancements aided the discovery of novel regulatory small RNAs called “isomiRs,” the canonical variants of miRNAs (Jeong et al. 2013). These multiple miRNA isoforms/isomiRs are usually generated from a single locus by DICER from imprecise cleavage, which is perfectly complementary to their pre-miRNA sequences. IsomiRs differ from the canonical miRNAs by nucleotide variation in their 5′ or 3′-end or both of the seed regions thus targeting a different mRNA molecule. Based on the variations in length, isomiRs are classified as 5′, 3′, and polymorphic isomiRs. Comparatively, the existence of 3′ substituted isomiRs is more evident in plants than 5′ substituted isomiRs, which are considered as a potential source for target site alterations (Balyan et al. 2020; Yang et al. 2019). In plants, the first isomiRs were reported in rice followed by peach, Arabidopsis, common bean, etc., and their decisive role in plant development and stress response also has been elucidated (Jeong et al. 2013; Yang et al. 2019). IsomiRs regulates post-transcriptional responses by acting as canonical partners to miRNAs. These isoforms proved to be functionally capable of target cleavage and thereby involved in the miRNA regulatory network. Studies suggested that canonical miRNA variants and their targets are evolutionarily conserved and are lineage-specific. For instance, miR156 is one of the broad and highly conserved miRNA family domains, and its regulation has been widely documented in plants that comprise different isoforms (Yang et al. 2019). Studying various isoforms of different miRNAs and lineage-specific isomiRs with respect to their parent miRNAs would help to understand their similar or differential roles in development, and stress responses (Budak et al. 2015; Yang et al. 2019).

## Regulation of ncRNAs synthesis and decay

Besides the synthesis, the decay of the ncRNAs is equally an essential process to maintain homeostasis. Moreover, this additional layer of modulating ncRNA expression, processing, and action will provide plasticity to the roles played by ncRNAs. Regulation of production and decay of different ncRNAs is still not completely understood in plants except for miRNAs with a few examples. In Arabidopsis, cyclin-dependent kinase F: 1 (CDKF: 1) controls the transcription of *MIR* genes by regulating Pol II activity by phosphorylation. Similarly, a conserved transcriptional co-activator (a multi-subunit complex) reduces the loading of Pol II at *MIR* gene promoters (Hajheidari et al. 2012). Negative on TATA less2B (NOT2b) protein interacts with the Pol II for the efficient transcription of *MIR* genes (Wang et al. 2019a). Similarly, the cell division cycle 5 (CDC5) protein functions as a positive regulator of transcription in association with both Pol II and *MIR* promoters (Wang et al. 2019a). Apart from the transcription process, subsequent steps of biogenesis were also regulated. SE, dsRNA-binding protein, HYL1, and TOUGH (TGH) proteins interact with DCL1, to regulate miRNA accumulation. Similarly, feedback regulation of miRNAs biogenesis is a well-known phenomenon, where the miRNAs regulate their own biogenesis. miR162 and miR168 are the two key feedback regulatory miRNAs by targeting *DCL1* and *AGO1* mRNAs (Song et al. 2019; Wang et al. 2019a). Furthermore, methylation plays a prominent role in stabilizing and destabilizing the miRNAs. Usually, unmethylated miRNAs can be easily degraded either by uridylation-dependent or by independent mechanisms. Methylated miRNAs can also be degraded through an unknown mechanism followed by the uridylation process. Overexpression of HUA ENHANCER1 (HEN1) SUPPRESSOR1 (HESO1) reduces miRNA accumulation in *hen1* mutants of Arabidopsis (Song et al. 2019). Small RNA degrading nuclease (SDN) also plays an important role in the turnover of mature miRNAs, and its deactivation results in the accumulation of miRNAs and diminished plant development (Xie et al. 2015). More extensive studies are required to understand the network of proteins/enzymes involved in the regulation of synthesis and decay of miRNAs and other ncRNAs.

## Role of regulatory ncRNAs in plant growth and development

The crucial role of regulatory ncRNAs in plant growth and development has been elucidated in detail in many reviews and research articles. Especially, there are many studies on miRNAs and lncRNAs describing their regulatory role in plant growth and development (Li and Zhang 2016; Swarup and Denyer 2018; Yu et al. 2019). Here, we primarily discussed how these ncRNAs regulate different complex networks. Different phase transitions from seed germination-vegetative-reproductive stages are crucial in the plant development process besides the growth of different tissues and organs.

The role of miRNAs as gene regulators in plant growth and development has been demonstrated in several studies (Li and Zhang 2016; Swarup and Denyer 2018; Yu et al. 2019). Majorly, plant miRNAs target different transcription factors (TFs) and genes of various pathways to regulate diverse biological processes (Fig. 4). Efforts to understand the regulatory mechanisms in controlling different plant developmental stages have led to the discovery of numerous miRNAs and their complex gene networks (Das et al. 2015; Swarup and Denyer 2018). Different miRNA-target modules especially, miR156-*SQUAMOSA PROMOTER BINDING PROTEIN-LIKE* (*SPL)*, miR159*-MYELOBLASTOSIS (MYB)*, and miR172-*APETALA 2* (*AP2*) are the key regulators in different plant developmental phase transitions (Ma et al. 2020; Swarup and Denyer 2018). These modules act either as positive or as negative regulators in promoting from one phase to another. For instance, in Arabidospsis, miR156-*SPL* module acts as a negative regulator for germination-vegetative-reproductive stage transitions, where the decreased levels of miR156 elicit the *SPL* expression to accelerate the transitions. On the other hand, miR172-*AP2* module acts as a positive regulator for the same transition, where the increased levels of miR172 decrease the *AP2* expression and thus promotes transition. This clearly implicates that miRNAs can turn on and off the specific pathways by fine-tuning the expression of targets. Several studies showed that miRNAs establish the regulatory networks by coordinating with different hormones like gibberellic acid (GA) and abscisic acid (ABA) to control germination and dormancy processes in plants (Das et al. 2015; Liu and El-Kassaby 2017; Martin et al. 2010). For instance, miR159 plays a vital role in controlling seed dormancy and germination via altering the balance between ABA and GA hormones (Martin et al. 2010). miR159 regulates the expression of MYB TFs, MYB33, and MYB101 to establish a positive regulation through ABA signaling in seed germination and dormancy (Reyes and Chua 2007). Furthermore, multiple members of the same gene family targeted by discrete miRNAs result in diverse biological functions. For instance, miR160 controls seed germination by negatively regulating the expression of *AUXIN RESPONSE FACTORS* (*ARFs*) in rice, and Arabidopsis (Das et al. 2015). Similarly, miR167 controls the root development by modulating the expression of *ARF6*, and *ARF8* by positive regulation (Gleeson et al. 2014). In leaf, miR165/166 regulates polarity with miR390 by targeting several *ARF* genes through the production of siRNAs (Chitwood and Timmermans 2010). These findings suggest the discrete regulatory role of miRNAs in different developmental transitions by mediating definite signaling pathways. In addition, miRNAs also act in an integrative mode on a single biological function as discussed by Yu et al. (2019). Furthermore, isoforms of a miRNA family might participate in similar biological functions either through the same or through different target genes (Alptekin et al. 2017). For instance, miR159a.1-*MYB* and miR159a-p5-*TETRAKETIDE ALPHA-PYRONE REDUCTASE 1* (*TKPR1*) modules associated with male meiosis and were significantly expressed in pollen and embryo sac. Thus, the complex regulatory network of miRNA-target modules forms the molecular basis of growth and development.

Besides miRNAs, studies showed the role of siRNAs in plant development (Khraiwesh et al. 2012). The phased *TAS3*-tasiRNAs derived from miR390-AGO7 complex targets *ARF* family members, which regulate various biological processes. The *TAS*3-tasiRNAs-*ARF* regulatory network emerged as the most conserved module in plants by regulating developmental transitions, embryo development, root structure, shoot apical meristem (SAM) development, leaf morphology, and flower and phytohormone cross-talk (Deng et al. 2018; Xia et al. 2017; Yu et al. 2019). In Arabidopsis, *TAS4*-tasiRNAs are triggered by miR828 targeted *MYB* genes (*PRODUCTION OF ANTHOCYANIN PIGMENT 1* (*PAP1*), *PAP2*, and *MYB113*), which are involved in the regulation of anthocyanin biosynthesis pathway (Zhou et al. 2020). Two homologous *MYB* genes regulate fiber development in cotton, and interestingly, these regions were *TAS4* orthologs in Arabidopsis and cotton, where one of the MYB genes is targeted by miR828 to generate 21-nt phasiRNAs. Furthermore, miR828 derived *cis*, *trans* siRNAs and phasiRNAs also regulate trichome development (Guan et al. 2014). Besides these studies, the role of natsiRNAs and hcsiRNAs in plant development is comparatively less. The involvement of cell-specific natsiRNA in the double fertilization process by regulating KOKOPELLI (*KPL*) and *ARIADNE14 (ARI14*) genes has been studied in Arabidopsis (Borges and Martienssen 2015).

In addition to the sncRNAs, lncRNAs tend to play a significant role in different developmental processes of plants by regulating the expression of neighboring genes by acting in *cis* and distant genes by *trans* modes (Yu et al. 2019). The possible mechanisms of the regulation are by chromatin/histone modifications through recruiting proteins, by acting as miRNA mimics, transcriptional regulation, and silencing or post-translational modifications. For instance, lncRNAs acting in *cis* were identified in root growth regulation and flowering time of Arabidopsis. The expression of *PINOID* (*PID*), an auxin transport gene, is regulated by the long intergenic non-coding RNA (lincRNA) *AUXIN-REGULATED PROMOTER LOOP* (*APOLO*) through the formation of chromatin loop, thus regulating the root growth in Arabidopsis (Ariel et al. 2014). Similarly, the lncRNA, *COLD-INDUCED LONG ANTISENSE INTERGENIC NON-CODING RNA* (*COOLAIR*) regulates the expression of *FLOWERING LOCUS C* (*FLC*) gene through the association of chromatin and recruiting chromatin modifiers, and thus regulating flowering time and seed dormancy (Chen and Penfield 2018). Another example of *cis* acting lncRNA was *LAIR* (l-(*LEUCINE-RICH REPEAT RECEPTOR KINASE* (*LRK*)) *ANTISENSE INTERGENIC RNA*), which regulates grain yield in rice by recruiting chromatin-modifying complexes to increase H3K4me3 and H4K16ac levels of its target *LRK* gene (Wang et al. 2018a). Similarly, few examples of *trans* acting lncRNAs were *ALTERNATIVE SPLICING COMPETITOR* (*ASCO*), which regulates the *NUCLEAR SPECKLE RNA-BINDING* (NSR) mRNA by modulating the alternative splicing patterns during root development of Arabidopsis (Bardou et al. 2014). Also, another lincRNA, *HIDDEN TREASURE 1* (*HID1*) promotes photomorphogenesis and represses the greenness of cotyledons by regulating the expression of *PHYTOCHROME-INTERACTING FACTOR 3* (*PIF3*) through chromatin interaction (Wang et al. 2014). Furthermore, increased expression of lncRNA, *long-day-specific male-fertility-associated RNA* (*LDMAR*), is essential for the pollen development during long-day conditions and single-nucleotide polymorphism (SNP) at the *LDMAR* locus increases RdDM at its promoter region and reduces *LDMAR* transcription (Ding et al. 2012).

Furthermore, studies on understanding the role of circRNAs in plant development have also been documented (Chu et al. 2018; Liu et al. 2017a; Zhang et al. 2020; Zhao et al. 2019). In Arabidopsis, increased expression of circRNAs associated with porphyrin, chlorophyll metabolism, and signal transduction of hormones was detected during leaf senescence (Liu et al. 2017a). In another study, a circRNA derived from the 6th exon of *SEPALLATA3* (*SEP3*) negatively regulates its own gene by acting in *cis* by binding to its cognate DNA locus and forming R-loop. This results in transcriptional pausing and increased abundance of alternative splice variants of the parental transcript (*SEP3*), which in turn results in the surge of floral homeotic phenotypes (Conn et al. 2017). The overexpression of rice circRNA, Os08circ16564, resulted in a severe decline of the *AK064900* gene, which has been involved in the development of rice spikelet and floral organs (Lu et al. 2015). Though sequencing technologies aid the identification of several circRNAs in plants, the regulation of circRNAs still needs more in-depth studies.

In addition, discoveries of other ncRNAs have added a piece of interesting information in this area of research (Cho 2018; Cho and Paszkowski 2017; Martinez et al. 2017). The tRNA-derived ncRNA, tRFGlu-CTC-5A, showed specific expression in flower tissues, while 5′-tRFs were accumulated in pollen tissue of Arabidopsis (Martinez et al. 2017). Expression of TE-derived lncRNA called *MIKKI* was detected in rice roots, and it has multiple intron sites that produce a binding site for miR171 upon splicing (Cho and Paszkowski 2017). Despite appreciable success in understanding the role of miRNAs in plant development, the functions and biological mechanisms of emerging ncRNAs like circRNAs and derived ncRNAs are still unclear. Intensive efforts are needed to ascertain the functional and regulatory role of emerging ncRNAs in concert to coordinate the different biological functions and mechanisms during plant development.

## Role of regulatory ncRNAs in plant stress responses

Regulatory roles of ncRNAs in various stress episodes also have been well-studied in plants. Activation of different regulatory ncRNAs by biotic and abiotic stress elicitors leads to the regulation of crucial stress-responsive pathways through target transcripts (Fig. 5). As stated in the earlier section, among different ncRNAs, miRNAs are the extensively investigated class followed by siRNAs and lncRNAs (Khraiwesh et al. 2012; Song et al. 2019; Yu et al. 2019). Regulation of gene expression mediated by miRNAs during different stress responses (drought, heat, salinity, cold, nutrient, and pathogen) has been exemplified in a different model and crop plants such as Arabidopsis, wheat, rice, maize, and barley (Barciszewska-Pacak et al. 2015; Ferdous et al. 2017; He et al. 2019; Hua et al. 2019; Mangrauthia et al. 2017a; Sailaja et al. 2014). In addition, there are comprehensive reviews that delineated the expression and regulation of different conserved miRNAs during various environmental stress episodes (Ferdous et al. 2015; Megha et al. 2018; Song et al. 2019; Zhao et al. 2016).

The wide range of miRNAs expression in each stress response has been witnessed in many plant species. However, few miRNA-target modules can show definite expression patterns against specific stress by regulating target genes, and their pattern of expression can be conserved across different plant species (Song et al. 2019). In addition to plant growth and development, the role of conserved miRNA-target modules is also crucial in conferring stress tolerance by integrating with metabolic pathways. Well-known conserved miRNA-target modules such as miR156-*SPL*, miR159-*MYB*, miR160-*ARF*, miR164-*NAC* (*NAM*, *ATAF*, and *CUC*), miR167-*ARF*, miR169-*NUCLEAR TRANSCRIPTION FACTOR-Y* (*NFY*), miR319-*TEOSINTE BRANCHED/CYCLOIDEA/PROLIFERATING CELL FACTORS* (*PCF*) (*TCP*), miR394-*LEAF CURLING RESPONSIVENESS* (*LCR*), miR396-*GROWTH REGULATING FACTOR* (*GRF*), and miR398- *COPPER/ZINC SUPEROXIDE DISMUTASE* (*CSD*) are known to play an important regulatory role in different stress environments to mitigate the detrimental effects (Fig. 4). For instance, different miRNAs are known to target TFs in phytohormone regulation, such as ABA, GA, ethylene signaling, and auxin signaling under drought conditions (Ferdous et al. 2017). The miR167-*ARF* module regulates the auxin signaling pathway during drought stress. *ARF6* and *ARF8*, the targets of miR167, negatively regulates auxin signaling pathway through *GRETCHEN HAGEN 3* (*GH3*). During drought, miR167 was upregulated in Arabidopsis, wheat, and maize, while it was downregulated in rice (Song et al. 2019). Similarly, miR169-*NFY* module also plays a significant role during water-deficit conditions. In Arabidopsis, tomato, and Medicago, downregulation of miR169 enhances the expression of its target, *NF-Y* (Li et al. 2008; Megha et al. 2018; Zhang et al. 2011a). The increased expression of *NF-Y* in stomatal guard cells enhances the drought tolerance by controlling the aperture of the guard cell in plants (Li et al. 2008). Besides, miR160-*ARF*, miR156-*SPL*, miR159-*MYB33*, miR164-*NAC*, miR172-*AP2* etc. modules are also shown to be involved in the regulation of drought stress response (Song et al. 2019). Similarly, several miRNAs were also identified in the regulation of plant’s heat stress response (Mangrauthia et al. 2017a; Ravichandran et al. 2019; Sailaja et al. 2014; Wang et al. 2011). In Arabidopsis, Brassica, and Populus, one of the important and most conserved miRNA-target modules as a part of the heat stress response is miR398-*CSD* (Guan et al. 2013; Yu et al. 2012). In Arabidopsis, increased expression of miR398 enhanced the heat tolerance in plants by negatively regulating the expression of its targets — *CSD1*, *CSD2*, and *COPPER CHAPERONE OF CSD* (*CCD*) (Guan et al. 2013; Lu et al. 2013). Decreased levels of *CSD1*, *CSD2*, and *CCD* aids in the accumulation of heat shock transcription factors (HSFs) and heat shock proteins (HSPs). Furthermore, other conserved modules, viz., miR156-*SPL*, miR172-*AP2* also contribute to heat stress response in plants (Song et al. 2019; Zhao et al. 2016). In addition, the highly conserved miR394-*LCR* module participates in the cold stress response of plants. In Arabidopsis, overexpressed miR394a plants exhibit cold tolerance by negatively regulating the *LCR* gene (Song et al. 2016). Furthermore, the increased expression of genes encoding *C-REPEAT BINDING FACTORS* (*CBFs*) or *DEHYDRATION-RESPONSIVE ELEMENT-BINDING FACTORS 1* (*DREB1*) in overexpressed miR394 and *lcr* mutant plants exhibits cold stress tolerance, which infers the positive regulation of miR394 through CBF-dependent pathway in acquiring cold stress tolerance (Song et al. 2016). Furthermore, the regulatory role of conserved miR319-*TCP* module conferring salinity tolerance in plants evidenced through overexpression studies of osa-miR319a in bentgrass (*Agrostis stolonifera*) (Zhou et al. 2013). In addition to the abovementioned studies, the involvement of miRNAs in nutrient uptake and nutrient homeostasis also has been shown. For instance, participation of miR399-*PHOSPHATE OVER ACCUMULATOR 2* (*PHO2*) module during phosphate deficiency, miR827-*NITROGEN LIMITATION ADAPTATION* (*NLA*) and miR169-*NF-Y* modules in nitrogen deficiency, and miR395-*SULFATE TRANSPORTER2;1* (*SULTR2*) in sulfur assimilation and transportation were also studied (Song et al. 2019). Besides miRNAs, studies also suggested the role of different isomiRs in plant stress responses. For instance, the differential expression of various isomiRs of the conserved miR156 family was identified during drought stress in maize (miR156a, b, c, d, e, h, i, and l) and rice (miR156d-5p.2, miR156f-5p.2, miR156h-5p.2, and miR156j-5p.2) (Balyan et al. 2020; Zheng et al. 2019a). Also, during heat stress, the highly differential expression of miR156 isoform than its canonical miRNA has been witnessed in Arabidopsis, which elucidates the important regulatory role of isomiRs (Baev et al. 2014).

Furthermore, the regulatory role of miRNA-target modules during biotic stresses caused by bacteria, fungi, viruses, and insects has also been established (Brant and Budak 2018; Khraiwesh et al. 2012; Song et al. 2019) (Fig. 4). In Arabidopsis, the regulatory role of miR393-*TRANSPORT INHIBITOR RESPONSE1*(*TIR1*), *AUXIN SIGNALING F-BOX1* (*AFB2*), and *AFB3* was the first identified module as a defensive response against *Pseudomonas syringae* pv*. tomato* DC3000, a bacterial pathogen. Here, increased miR393 expression levels due to bacterial PATHOGEN-ASSOCIATED MOLECULAR PATTERNS (PAMP) flagellin (flg22) downregulate *TIR1*, *AFB2*, and *AFB3*, which results in increased bacterial resistance. Similarly, pathogen-associated triggered immunity in response to fungal pathogens, miR773-*METHYLTRANSFERASE 2* (*MET*2) module, displayed enhanced resistance (Salvador-Guirao et al. 2018). Also, in rice, the miR528-*ASCORBATE OXIDASE* (*AO*) module contributes towards the enhancement of viral defense by accumulating reactive oxygen species (ROS). Upon the rice stripe virus (RSV) infection, miR528 masked by AGO 18 leads to elevated *AO* activity and in turn helps in the accumulation of basal reactive oxygen species (ROS) to enhance antiviral defense. In addition to the above discussed prominent regulatory roles of miRNA modules in both abiotic and biotic stress responses, there are many other modules (reviewed in Song et al. 2019) and are not further discussed here.

In addition, other sncRNAs like tasiRNAs are also shown to be involved in plant stress responses. For instance, *HEAT-INDUCED TAS1 TARGET 1* (*HTT 1*) and *HTT 2* mRNA targets of *TAS1* (*trans*-acting siRNA precursor 1)-derived tasiRNAs form miR173 contribute to thermotolerance in Arabidopsis (Li et al. 2014a). Plants with elevated levels of *TAS1*-siRNAs and decreased levels of the *HTT* genes are sensitive to heat stress, while the plants overexpressing *HTT1* and *HTT2* genes exhibited enhanced thermotolerance (Li et al. 2014a). Furthermore, during phosphate homeostasis, positive regulation of protein derived from *PHOSPHATE1;2* (*PHO1;2*) gene and its *cis*-NAT (*cis*-NAT_*PHO1;2*_) in Arabidopsis has been confirmed. Downregulation of *cis*-NAT_*PHO1;2*_ through RNAi revealed the impaired allocation of phosphate from root to shoot, which ultimately led to reduced seed yield by reduction of PHO1;2 proteins (Jabnoune et al. 2013). Similarly, the regulatory role of natsiRNAs during salt stress was demonstrated in Arabidopsis. natsiRNA (24 nt) generated from S*IMILAR-TO-RCD-ONE 5* (*SRO5*) mRNA, targets *D1-PYRROLINE-5-CARBOXYLATE DEHYDROGENASE* (*P5CDH*) results in the subsequent formation of 21 nt natsiRNAs. The generated natsiRNAs further participates in the cleavage of *P5CDH* mRNA. During salt stress, induction of SRO5 protein results in the declined expression of *P5CDH* activity leading to proline and reactive oxygen species (ROS) accumulation. Thus, the role of natsiRNAs of *SRO5* on *P5CDH* genes, together with their respective proteins in osmoprotection and oxidative stress during salt stress has been confirmed (Borsani et al. 2005; Khraiwesh et al. 2012). Similarly, the role of phasiRNAs derived from miR482, miR828, and miR6455 during drought stress was studied in populus, where populus-specific miR6455 derived 22-nt phasiRNA targeted *NAC* genes, that are known to play a crucial role in drought stress (Shuai et al. 2016). Furthermore, during biotic stress, the first plant-endogenous siRNA *nat-siRNAATGB2* regulates *R-gene*-mediated ETI (effector-triggered immunity) towards bacterial pathogen *Pseudomonas syringae* (*Ps*) infection (Navarro et al. 2006). Induction of this siRNA inhibits the expression of antisense target *PENTATRICOPEPTIDE REPEAT PROTEIN-LIKE* (*PPRL*), a negative regulator of *RPS2*-mediated ETI in response to *Ps*. Generated endogenous siRNA, *nat-siRNAATGB2*, aids in *R-gene*, *RPS2*-mediated race-specific disease resistance by inhibiting the expression of predicted negative regulator *PPRL* gene (Katiyar-Agarwal and Jin 2010). Furthermore, in Arabidopsis, phasiRNAs derived from *PPR* genes confers a defensive response against the *Phytophthora capsici* infection (Hou et al. 2019). In tomato, transgenic lines expressing short tandem target mimic (STTM) RNAs of miR482/2118 confirm the role of derived phasiRNAs in the regulation of *nucleotide-binding site leucine-rich repeat* (*NLR*) genes and the important role of NLR proteins in conferring disease resistance against bacterial and oomycete pathogens (Canto-Pastor et al. 2019). Similarly, overexpression of two tasiRNAs derived from *TAS1* and *TAS2* loci resulted in reduced virulence against the fungal pathogen *Botrytis cinerea* (Cai et al. 2018). Also, a study by Wu et al. (2020) reported the crucial role of 22-nt siRNAs derived from *nitrate reductase* (*NIA1* and *NIA2*) genes helps in plant adaptation to different environmental stress responses by inducing gene silencing and translational repression. In addition to the mechanistic theme of regulation by sncRNAs, the emerging lncRNAs also have considerable attention for their regulatory role in plant stress responses.

lncRNAs that are responsive to different abiotic and biotic stresses also have been identified in different plant species. For instance, drought-responsive lncRNAs have been identified in Arabidopsis, populus, maize, rice, etc., (Chung et al. 2016; Di et al. 2014; Pang et al. 2019; Qin et al. 2017; Shuai et al. 2014). During stress periods, it is evident that lncRNAs regulate the expression of multiple genes through possible mechanisms and act as potential gene regulators in different plant biological processes. For instance, in Arabidopsis, the lncRNA, *DROUGHT INDUCED LNCRNA* (*DIR*) is responsive to drought and salinity stress and acts as a positive regulator by modifying the expression of a series of genes. The overexpressed *DIR* plants exhibited enhanced drought and salinity tolerance (Qin et al. 2017). In rice, genes encoding for zinc-finger proteins of drought QTL region, *qSDT2-1*, were found to be the predicted targets of identified lncRNAs, which signifies their regulatory role in drought stress (Weidong et al. 2020). Similarly, heat stress–responsive lncRNAs were also identified in brassica, cassava, rice, etc., (Ding et al. 2019; Luo et al. 2018; Wang et al. 2019b). In *Brassica rapa*, two heat stress–responsive lncRNAs identified as endogenous target mimics for miR164a and contrasting expression of both miRNA and lncRNA define their important role in heat stress response (Wang et al. 2019b). Furthermore, different abiotic stress–responsive lncRNAs act as target mimics for miR156, miR159, and miR172, thus involves in the regulation of various stress-responsive genes ABA, ethylene signaling, HSPs, and HSFs pathways (Ding et al. 2019; Wang et al. 2019b). Similarly, cold and salinity–responsive lncRNAs were identified in several plant species (Karlik and Gozukirmizi 2018; Qin et al. 2017; Wang et al. 2015b, 2019c). Two lncRNAs, *COOLAIR* and *COLD ASSISTED INTRONIC NON-CODING RNA* (*COLDAIR*), promote flowering in plants during cold conditions (Whittaker and Dean 2017). Similarly, signatures of lncRNA regulation in biotic stress responses were evident from different studies (Nejat and Mantri 2017; Yu et al. 2019). For instance, in tomato during *Phytophthora infestans* infection, the lncRNA16397 induces the expression of *GLUTAREDOXIN 22* gene by acting in *cis* and resulted in the enhanced resistance (Cui et al. 2017). Collectively, these results demonstrate the complex regulatory function of lncRNAs in defensive pathways by modulating the expression of defense responsive genes.

Similarly, the research on stress-responsive circRNAs and derived ncRNAs is in the course of its way. The expression of stress-responsive circRNAs using high-throughput sequencing technologies has been identified. In wheat, Wang et al. (2016a) identified 62 circRNAs in response to dehydration stress. Similarly, in pear fruits, 23 circRNAs showed increased expression during drought stress (Wang et al. 2018b). Furthermore, the expression of circRNAs in response to bacterial pathogen infection by *Pseudomonas syringae* pv. *actinidiae* (*PSA*) in kiwi fruits and by *Pectobacterium carotovorum* subsp. *Brasiliense* (*PCB*) infection in potato delineates their role in biotic stress (Wang et al. 2017a; Zhou et al. 2018). A recent study by Fan et al. (2020) in rice showed the contribution of circRNAs in response to *Magnaporthe oryzae*, a fungal pathogen. The high diversity of circRNAs with tolerant genotype (IR25) during *M. oryzae* infection is due to more 3′ and 5′ alternative back-splicing and complex splice sites. Furthermore, the role of circR5g05160 in enhancing immunity against *M. oryzae* has been reported (Fan et al. 2020). Besides circRNAs, accumulation of different derived ncRNAs such as tRFs (tRNA-Val-CAC, tRNA-Thr-UGU, tRNA-Tyr-GUA, and tRNA-Ser-UG) has been reported during heat and osmotic stress in wheat and phosphate stress in Arabidopsis and barley (Hackenberg et al. 2013; Hsieh et al. 2010; Wang et al. 2016b). Furthermore, activation of TE-derived lncRNA11195 after various abiotic stress treatments in Arabidopsis revealed the important role of transposon-derived lncRNAs in stress responses (Wang et al. 2017b). Though sequencing technologies expedite our understanding on the circular and derived ncRNAs in plants, still their functional characterization and in-depth investigation are prerequisite to assign the exact role of these emerging regulatory non-coding RNAs. We have summarized the stress-responsive regulatory non-coding RNAs and their expression which are valuable molecular resources in Tables S1 and S2, to understand their regulatory patterns associated with stress tolerance and plant defense mechanisms.

## Harnessing the regulatory ncRNAs for crop improvement

Utilizing the available ncRNAs’ information and their regulation would be a desirable application to address food and nutritional security. To use the ncRNAs’ information in the improvisation of key traits in various crops, different molecular genetics–based approaches have been employed. There are some classic reports which demonstrate the effect of single miRNA manipulation for diverse traits such as increased crop yield, biomass, and stress tolerance (Zhang and Wang 2016). For instance, overexpression of evolutionarily conserved miR156 in tomato plants showed association with fruit size (Zhang et al. 2016b). The *SPL* genes, targets of miR156, showed a positive association with rice yield (Jiao et al. 2010; Wang et al. 2012). In rice, miR156 regulates the expression of *SPL13*, *SPL14*, and *SPL16* genes that in turn govern the regulation of grain size and panicle (Jiao et al. 2010; Tang and Chu 2017; Wang et al. 2012). Similarly, increased expression of miR397 showed a positive correlation with the grain size and yield by regulating its target gene, *LACCASE* (*LAC*) in rice (Zhang et al. 2013a). Wang et al. (2016c) reported enhanced expression of miR444 in rice, which resulted in the downregulation of *MAD23*, *MAD27a*, and *MADS57* during RSV infection, through activating RDR1-dependent antiviral RNA-silencing pathway.

Further, artificial miRNA (amiRNA/amiR) has also been suggested as a potential approach for crop improvement by constructing amiRNAs to regulate the target gene expression (Rosa et al. 2018; Zhang et al. 2018a). One such example is the construction of amiR159b to target three crucial genes involved in seed oil metabolism, viz., *fatty acid Δ*^*12*^* desaturase 1* (*FAD2*), *fatty acid elongase 1* (*FAE1*), and *fatty acyl-ACP thioesterase B* (*FATB*) for high oleic content in Arabidopsis (Belide et al. 2012). The ability of amiR to target multiple traits is an added advantage to this approach. For instance, Ai et al. (2011) showed that co-expression of various amiRNAs targeting different viruses in transgenic plants leads to multiple virus resistance (Ai et al. 2011). amiRNAs designed from miR159a, miR167b, and miR171a precursors of Arabidopsis targeting expression of suppressor HC-PRO and P25 confer resistance towards *Potato virus Y* (*PVY*) and *Potato virus X* (*PVX*), respectively.

From markers’ perspective, single-nucleotide polymorphisms (SNPs) are present abundantly in ncRNA regions which is an essential feature in crop improvement as prospective biomarkers (Fabbri et al. 2019). The base composition for miRNA/premiR sequence is very crucial for its function and the altered bases/SNPs in the premiR sequence resulted in the unstable secondary structure. Some of the important agronomic traits like grain length and seed type differentiation were found to be associated with SNPs in premiR of miR2923a (Wang et al. 2013). These variations in ncRNAs can be further exploited to improve agronomic traits of interest. Similarly, the *PHOTOPERIOD-SENSITIVE GENIC MALE STERILITY 1* (*Pms1*) locus encodes lncRNA, *PMS1T*, specifically expressed in young panicles of rice (Fan et al. 2016). miR2118 targets *PMS1T* to produce 21-nt phasiRNAs that specifically accumulated in the male sterile line during long-day conditions. SNP in *PMS1T* nearby miR2118 recognition site suggest its possible mechanism in reproductive development of rice. Availability of whole genome sequences, followed by resequencing of multiple lines for a given crop species, provides an opportunity to look into the natural variations existing in the genome. Combining such information with the transcriptomic studies may aid in the identification of the functional role of such variations mediated through ncRNAs.

In addition, studies showed that genome editing with site-specific nucleases, especially with type II clustered regularly interspaced short palindromic repeats (CRISPR)/CRISPR-associated protein 9 (Cas9) system, is the powerful genome editing approach for functional studies in plants (Basso et al. 2019; Chen et al. 2019; Mangrauthia et al. 2017b). CRISPR/Cas9 has shown the possibility to overcome the limitations associated with RNAi by contributing to complete gene knockout and reduced off-target activities (Basso et al. 2019). Localization of certain ncRNAs in the nucleus possesses challenges with the implementation of RNAi as it is limited to the cytoplasm, where RISC is located. In the case of plants, the application of genome editing in crop improvement by targeting ncRNAs is in the emerging stage and efforts are being made to use the system efficiently (Basso et al. 2019). For instance, Jacobs et al. (2015) targeted two miRNAs, miR1514 and miR1509, in soybean with Cas9 and demonstrated the strong potential of targeting short ncRNA as a target using CRISPR/Cas9 (Jacobs et al. 2015). In a similar way, by using the CRISPR/Cas9 non-homologous end joining (NHEJ) strategy, one can introduce indels at pre-miRNA sequences or the miRNA processing sites of *MIR* genes to regulate miRNA biogenesis (Zhou et al. 2017). Collectively, the abovementioned precision technologies would help in the effective utilization of ncRNAs’ information in crop improvement by developing cultivars with desirable characteristics.

This review on diverse ncRNAs ranging from the most familiar (miRNAs, siRNAs) to the emerging ncRNAs (lncRNAs, circRNAs, tRFs, and rDNA-derived miRNAs) and their isovariants identified in different plant species provides a better understanding of their functions at multiple stages of transcriptional and post-transcriptional gene regulation. Of note, the intervention of ncRNAs in the epigenetic mechanisms highlights their potential, leading to genotypic/phenotypic variations across the plant species. We speculate that discussed ncRNAs were having their own decisive role in governing the regulation of plant growth, development, and environmental stress responses. The basic understanding of the characteristic features and functions of heterologous ncRNA species helps to link ncRNA function to the specific plant trait. It also highlights the crucial role of various ncRNAs, including target genes and their expression profiles under biotic and abiotic stresses, which will facilitate the trait-specific ncRNA selection and its deployment in crop engineering.

## Conclusions and future perspectives

This review expands our knowledge about the intertwined regulatory role of ncRNAs. With the advent of deep sequencing technologies, the identification of diverse ncRNAs and their profiling is increasing at unprecedented levels. In this context, we attempted to articulate the classification, biogenesis, and regulatory roles of the available plant ncRNAs. Moreover, deep-diving into ncRNA biogenesis is essential, as the knowledge of proteins/enzymes which are controlling the expression/decay of ncRNA is meager. In short, it was suggested that biogenesis of these diverse ncRNAs is induced, as per the plant developmental needs and stress challenges. To overcome the challenges of food security, functional validation of key ncRNAs and their isoforms has been piloted through the overexpression or knockout studies of siRNA/miRNAs. Of note, for the accurate view of ncRNA function, a novel tool CRISPR-Cas can be exclusively applied to prevent potential off-target mutations. So far, the functional studies on ncRNAs in plants confine mostly to miRNAs in different perspectives like development and stress-responsive regulation. On the other hand, research on other ncRNAs is still at infancy, and more intensive efforts are needed to unravel the complexity and functional role of different ncRNAs, especially circRNAs and derived RNAs. Likewise, it is still astonishing to believe how plants synchronize the accumulation of these diverse ncRNAs as per their need. Furthermore, to utilize the information of ncRNAs for crop improvement, an extensive knowledge is essential to understand their functional and regulatory role in different gene regulatory networks. In addition, it is imperative to develop a trait-specific candidate ncRNA catalogue which will be targets for engineering new crop varieties. Overall, this review will be helpful to the researchers to enhance their understanding of different classes of ncRNAs and their functional link with the plant phenotype and regulation. Though many challenges are yet to be addressed, strategic implementations of ncRNA-based approaches in molecular crop breeding will further strengthen to overcome the impending food crisis.

## Supplementary Information

Below is the link to the electronic supplementary material.

Supplementary file1 (DOC 825 KB)
