# Supplementary material for: Regulatory non-coding RNAs: a new frontier in regulation of plant biology
Source: Funct Integr Genomics. 2021 May 20;21(3-4):313–30. doi: 10.1007/s10142-021-00787-8 (PMC8298231; doi:10.1007/s10142-021-00787-8)
Supplement: Supplementary file 1 — Supplementary file1 (DOC 825 KB) [file 10142_2021_787_MOESM1_ESM.doc]

**­­­­­Regulatory non-coding RNAs: A new frontier in regulation of plant biology**

Sailaja Bhogireddy^1^,*, Satendra K Mangrauthia^2^, Rakesh Kumar^1,3^, Arun K Pandey^1,4^, Sadhana Singh^1^, Ankit Jain^1^, Hikmet Budak^5^, Rajeev K Varshney^1,6^ and Himabindu Kudapa^1^,*

^1^ Center of Excellence in Genomics & Systems Biology (CEGSB), International Crops Research Institute for the Semi-Arid Tropics (ICRISAT), Hyderabad, India

^2^ Crop Improvement Section, ICAR-Indian Institute of Rice Research, Hyderabad, India

^3^Department of Life Sciences, Central University of Karnataka, Karnataka, India

^4^ College of Life Sciences, China Jiliang University, Hangzhou, China

^5^ Montana BioAgriculture, Inc., Missoula, MT, United States

^6^ State Agricultural Biotechnology Centre, Centre for Crop and Food Innovation, Murdoch University,

Murdoch, Western Australia, Australia

*Corresponding authors: [sailajaprasadd@gmail.com](mailto:sailajaprasadd@gmail.com); [k.himabindu@cgiar.org](mailto:k.himabindu@cgiar.org)

**­**

**Table S1 Differential** expression patterns of non-coding RNAs in abiotic stress responses and their potential targets and functions in major plant species

| **ncRNA** | **Crop^a^** | **Stress Response^bc^** | | | **Target** | **Function** | **Reference** |
| --- | --- | --- | --- | --- | --- | --- | --- |
|  |  | **D** | **H** | **S** |  |  |  |
| **miRNAs** | | | | | | | |
| miR156 | *A. thaliana* |  |  |  | *Squamosa promoter*  *binding protein-like* (*SPL*) | Floral transition phase, abiotic stress response | Barciszewska-Pacak et al. 2015; Ishka et al. 2018; Stief et al. 2014; Zheng et al. 2019b |
|  | *T. aestivum* |  |  |  |  |  | Akdogan et al. 2016; Feng et al. 2017; Kumar et al. 2015 |
|  | *O. sativa* |  |  |  |  |  | Cui et al. 2014; Mangrauthia et al. 2017a |
|  | *Z. mays* |  |  |  |  |  | Ding et al. 2009; Liu et al. 2019; Zhang et al. 2019 |
| miR159 | *A. thaliana* |  |  |  | *Myeloblastosis* (*MYB*) | Gibberellin signalling, Biotic stress response | Barciszewska-Pacak et al. 2015; Liu et al. 2008; Li et al. 2016b; Reyes and Chua 2007 |
|  | *T. aestivum* |  |  |  |  |  | Kumar et al. 2014; Lu et al. 2011; Wang et al. 2012b |
|  | *O. sativa* |  |  |  |  |  | Liu et al. 2017b; Parmar et al. 2020 |
|  | *Z. mays* |  |  | - |  |  | Aravind et al. 2017; He et al. 2019 |
| miR160 | *A. thaliana* | - |  |  | *Auxin response factors* (*ARF*) | Root, shoot and flower development, abiotic stress response | Barciszewska-Pacak et al. 2015; Lin et al. 2018 |
|  | *T. aestivum* | - |  |  |  |  | Feng et al. 2017; Kumar et al. 2015 |
|  | *O. sativa* |  |  | - |  |  | Bakhshi et al. 2016; Liu et al. 2017b |
|  | *Z. mays* |  |  |  |  |  | Aravind et al. 2017; Ding et al. 2009; Zhang et al. 2019 |
| miR162  Table S1. (Continued) | *A. thaliana* | - | - | - | *Dicer Like 1*(*DCL*) | miRNA processing | - |
|  | *T. aestivum* | - | - | - |  |  | - |
|  | *O. sativa* | - | - | - |  |  | - |
|  | *Z. mays* |  |  |  |  |  | Ding et al. 2009; He et al. 2019; Liu et al. 2019 |
| miR164 | *A. thaliana* |  |  |  | *NAC* | Lateral root development, early embryogenesis | Barciszewska-Pacak et al. 2015; May et al. 2013 |
|  | *T. aestivum* | - |  |  |  |  | Eren et al. 2015; Pandey et al. 2014 |
|  | *O. sativa* |  |  |  |  |  | Fang et al. 2014; Liu et al. 2017b; Macovei and Tuteja 2012; Zhang et al. 2016b |
|  | *Z. mays* |  |  |  |  |  | Ding et al. 2009; Fu et al. 2017; He et al. 2019; Liu et al. 2019 |
| miR166 | *A. thaliana* |  |  |  | *Homeodomain–leucine zipper* (*HD-Zip*), *REVOLUTA (REV), PHABULOSA (PHB), PHAVOLUTA (PHV), CORONA (CNA)* | Highly conserved and abundant miRNA, SAM development, seed development, organ polarity and nutrition ion uptake | Barciszewska-Pacak et al. 2015; Yan et al. 2016 |
|  | *T. aestivum* |  |  | - |  |  | Akdogan et al. 2016; Ravichandran et al. 2019 |
|  | *O. sativa* |  |  |  |  |  | Mangrauthia et al. 2017a; Parmar et al. 2020; Zhang et al. 2018b |
|  | *Z. mays* |  |  |  |  |  | He et al. 2019; Kong et al. 2010; Liu et al. 2019 |
| miR167 | *A. thaliana* |  |  |  | *Phospholipase D*,  *Auxin response factors* (*ARF*) | Auxin signalling ABA response and Nodule development, root plastic development, abiotic stress responses | Barciszewska-Pacak et al. 2015 |
|  | *T. aestivum* |  |  |  |  |  | Eren et al. 2015; Ravichandran et al. 2019 |
|  | *O. sativa* |  |  |  |  |  | Gleeson et al.2014; Paramar et al. 2020; Sailaja et al. 2014 |
|  | *Z. mays* |  |  |  |  |  | Fu et al. 2017; He et al 2019 |
| miR168  Table S1. (Continued) | *A. thaliana* |  |  |  | *Argonaute 1*(*AGO1*) | RISC loading in miRNA biogenesis and abiotic stress responses | Barciszewska-Pacak et al. 2015; Li et al. 2012 |
|  | *T. aestivum* | - |  |  |  |  | Gupta et al. 2014; Xin et al. 2010 |
|  | *O. sativa* |  |  | - |  |  | Gleeson et al. 2014; Liu et al. 2017b |
|  | *Z. mays* |  |  |  |  |  | Ding et al. 2009; He et al. 2019; Liu et al. 2019 |
| miR169 | *A. thaliana* |  |  |  | *Nuclear transcription*  *Factor Y subunit alpha*; (*NF-YA*) | ABA signalling, symbiotic nitrogen fixation, drought stress response | Barciszewska-Pacak et al. 2015; Liu et al.2008; Pegler et al. 2019 |
|  | *T. aestivum* |  |  |  |  |  | Akdogan et al. 2016; Feng et al. 2017; Raghupathy et al. 2016 |
|  | *O. sativa* |  |  |  |  |  | Paramar et al. 2020; Sailaja et al. 2014; Zhou et al. 2010 |
|  | *Z. mays* |  |  |  |  |  | Liu et al. 2019; Luan et al. 2014; Zhang et al. 2019 |
| miR172 | *A. thaliana* |  |  |  | *APETALA2* (*AP2*) | Temperature responsive flowering, nodule development | Lee et al. 2010; Li et al. 2016a |
|  | *T. aestivum* |  |  |  |  |  | Akdogan et al. 2016; Gupta et al. 2014 |
|  | *O. sativa* |  |  |  |  |  | Mangrauthia et al. 2017a; Paramar et al. 2020; Zhou et al. 2010 |
|  | *Z. mays* |  |  |  |  |  | Fu et al. 2017; Liu et al. 2019; Zhang et al. 2019 |
| miR319 | *A. thaliana* |  |  |  | *Teosinte Branched1* (*TCP*) | Abiotic stress response | Barciszewska-Pacak et al. 2015; Liu et al. 2008 |
|  | *T. aestivum* |  |  |  |  |  | Akdogan et al. 2016; Eren et al. 2015; Kumar et al. 2015 |
|  | *O. sativa* |  |  |  |  |  | Mangrauthia et al. 2017a; Parmar et al. 2020; Zhou et al. 2010 |
|  | *Z. mays* |  | - |  |  |  | Kong et al. 2010 |
| miR393  Table S1. (Continued) | *A. thaliana* |  |  |  | *Transport inhibitor*  *response 1* (*TIR1*)/ *Auxin signalling F-box* (*AFB*) | Auxin signalling | Lee et al. 2010; Liu et al. 2008; Sunkar and Zhu 2004 |
|  | *T. aestivum* | - |  |  |  |  | Gupta et al. 2014; Wang et al. 2014b; Xin et al. 2010 |
|  | *O. sativa* |  |  |  |  |  | Gao et al. 2011; Li et al. 2015; Zhao et al. 2007 |
|  | *Z. mays* | - | - | - |  |  | - |
| miR395 | *A. thaliana* |  |  | - | *ATP sulfurylase* and,  *Sulfate*  *transporter 2;1*  (*SULTR2;1*) genes | Nutrient stress response especially in Sulfate transport and assimilation | Jagadeeswaran et al. 2014; Pegler et al. 2019 |
|  | *T. aestivum* |  |  |  |  |  | Akdogan et al. 2015; Goswami et al. 2014; Kumar et al. 2015; Wang et al. 2014b |
|  | *O. sativa* |  | - |  |  |  | Khraiwesh et al. 2012; Shen et al. 2010; Zhou et al. 2010 |
|  | *Z. mays* |  | - |  |  |  | Ding et al. 2009; Liu et al. 2019 |
| miR396 | *A. thaliana* |  |  |  | *Growth-regulating*  *factor* (*GRF*), *basic helix-loop-helix* (*bHLH*) | Abiotic stress response | Barciszewska-Pacak et al. 2015; Liu et al. 2008 |
|  | *T. aestivum* |  |  |  |  |  | Akdogan et al. 2015; Wang et al. 2014b; Xin et al. 2010 |
|  | *O. sativa* |  |  |  |  |  | Gao et al. 2011; Li et al. 2015; Zhou et al. 2010 |
|  | *Z. mays* |  |  |  |  |  | Ding et al. 2009; He et al. 2019; Li et al. 2013 |
| miR397 | *A. thaliana* |  |  |  | *Laccase* (*Lac*) | Grain yield, cold stress response, lignin biosynthesis | Mahale et al. 2014; Sunkar and Zhu 2004 |
|  | *T. aestivum* | - |  |  |  |  | Wang et al. 2014b; Xin et al. 2010 |
|  | *O. sativa* |  |  | - |  |  | Sailaja et al. 2014; Zhou et al. 2010 |
|  | *Z. mays* |  | - | - |  |  | Liu et al. 2019 |
| miR398  Table S1. (Continued) | *A. thaliana* |  |  |  | *Cu/Zn superoxide*  *dismutases* (*CSDs*), *Copper*  *Chaperone of CSD* (*CCD)* | Heat stress response, oxidative stress response, and copper homeostasis | Barciszewska-Pacak et al. 2015; Guan et al. 2013; Jagadeeswaran et al. 2009 |
|  | *T. aestivum* |  |  |  |  |  | Akdogan et al. 2015; Ragupathy et al. 2016; Wang et al. 2014b |
|  | *O. sativa* | - |  | - |  |  | Sailaja et al. 2014 |
|  | *Z. mays* |  |  | - |  |  | He at al. 2019; Liu et al. 2019 |
| miR399 | *A. thaliana* |  | - |  | *Phosphate over accumulator 2* (*PHO2*); *Ubiquitin-conjugating Enzyme* (*UCB*) | Response to phospohate starvation | Barciszewska-Pacak et al. 2015 |
|  | *T. aestivum* |  |  |  |  |  | Akdogan et al. 2015; Xin et al. 2010; Wang et al. 2014b |
|  | *O. sativa* | - |  | - |  |  | Mangrauthia et al. 2017a |
|  | *Z. mays* |  |  | - |  |  | He at al. 2019; Liu et al. 2019 |
| miR408 | *A. thaliana* |  | - |  | *Lac*, *Plantacyanin* | Abiotic stress response, copper deficit response | Ma et al. 2015 |
|  | *T. aestivum* |  | - |  |  |  | Akdogan et al. 2015; Wang et al. 2014b |
|  | *O. sativa* |  |  | - |  |  | Mutum et al. 2013; Mangrauthia et al. 2017a |
|  | *Z. mays* |  |  | - |  |  | He at al. 2019; Liu et al. 2019 |
| miR474  Table S1. (Continued) | *A. thaliana* | - | - | - | *Protein kinase, kinesin, LRR, Proline dehydrogenase* | Proline accumulation | - |
|  | *T. aestivum* | - | - | - |  |  | - |
|  | *O. sativa* |  | - | - |  |  | Zhou et al. 2010 |
|  | *Z. mays* |  | - |  |  |  | Ding et al. 2009; Wei et al. 2009 |
| miR528 | *A. thaliana* | - | - | - | *Ascorbate Oxidase* (*AO*), *Lac*, *Copper ion* *binding* , *Red and far-red insensitive 2* | Promotes flowering, abiotic stress response | - |
|  | *T. aestivum* |  |  | - |  |  | Akdogan et al. 2015; Xin et al. 2010 |
|  | *O. sativa* | - |  | - |  |  | Mangrauthia et al. 2017a |
|  | *Z. mays* |  |  | - |  |  | He at al. 2019; Liu et al. 2019 |
| **LncRNAs** | | | | | | | |
| npc60 | *A. thaliana* | - | - |  |  | Salt stress responsive | Amor et al. 2009 |
| npc82 | *A. thaliana* | - | - |  |  | Salt stress responsive | Amor et al. 2009 |
| npc536 | *A. thaliana* | - | - |  |  | Salt stress responsive | Amor et al. 2009 |
| lnRNA5 | *T. aestivum* | - |  | - |  | Heat stress responsive | Xin et al. 2011 |
| lnRNA9 | *T. aestivum* | - |  | - |  |  | Xin et al. 2011 |
| lnRNA27 | *T. aestivum* | - | - | - |  |  | Xin et al. 2011 |
| TR24835 | *T. aestivum* |  |  |  | ND | Stress responsive | Sharma et al. 2017 |
| TR2184 | *T. aestivum* |  |  |  |  | Stress responsive | Sharma et al. 2017 |
| TR3125 | *T. aestivum* |  |  |  |  | Stress responsive | Sharma et al. 2017 |
| TR24194 | *T. aestivum* |  |  |  |  | Stress responsive | Sharma et al. 2017 |
| TR20678 | *T. aestivum* |  |  |  |  | Stress responsive | Sharma et al. 2017 |
| TR2158 | *T. aestivum* |  |  |  |  | Stress responsive | Sharma et al. 2017 |
| *asHSFB2a* | *A. thaliana* | - |  | - | *HSFB2a* | Heat stress response | Wunderlich et al. 2014 |
| *MSTRG69391* | *O. sativa* |  | - | - | ND | Drought stress response | Weidong et al. 2020 |
| *MSTRG62341* | *O. sativa* |  | - | - | *bZIP60* | Drought stress response | Weidong et al. 2020 |
| *TCONS_00028567* | *O. sativa* |  | - | - | *SAPK10 (Stress/ABA activated protein kinase 10)* | Drought stress response | Li et al. 2019 |
| *MSTRG.6838.1* | *Z. mays* |  | - | - | *VVP(vacuolar (H+ )-pumping ATPase)* | Drought stress response | Pang et al. 2019 |
| *Drought induced lncRNA* (*DRIR*) | *A. thaliana* |  | - |  | ND | Drought stress response | Qin et al. 2017 |

Table S1. (Continued)

**^a^Crop abbreviations:** ***A. thaliana****- Arabidopsis thaliana;* ***O. sativa****- Oryza sativa;* ***T. aestivum****- Triticum aestivum;* ***Z. mays****- Zea mays.*

**^b^Type of stress: D-** Drought; **H-** Heat; **S-** Salinity.

**^c^Symbols:** represents down-regulation; r represents up-regulation of small RNA species in the specified crop; ‘-‘ represents not determined.

**Table S2 Differential expression patterns of non-coding RNAs in biotic stress response and their potential targets and functions in different plant species**

| **ncRNA** | **Crop^a^** | **Pathogen** | **Stress Response^bc^** | | | **Target** | **Function** |
| --- | --- | --- | --- | --- | --- | --- | --- |
|  |  |  | **B** | **F** | **V** |  |  |
| **miRNAs** | | | | | | | |
| miR156 | *P. trichocarpa*  (Lu et al. 2007) | *C. quercuum f.sp. fusiform* |  |  |  | *Squamosa promoter*  *binding protein-like* (*SPL*) | Pathogen defence response |
| miR156 | *P. trichocarpa*  (Zhao et al. 2012) | *Botryosphaeria dothidea* |  |  |  | *SPL* | Pathogen defence response |
| miR156 | *A. thaliana*  (Li et al. 2010) | *Pseudomonas syringae*  *(P. syringae)* |  |  |  | *SPL* | Pathogen defence response |
| miR156 | *O. sativa*  (Wu et al. 2015) | *Rice strip virus* (*RSV*) |  |  |  | *SPL* | Pathogen defence response |
| miR159 | *A. thaliana*  (Zhang et al. 2011b) | *P. syringae* |  |  |  | *Myeloblastosis* (*MYB*) | Pathogen defence response |
| miR160 | *A. thaliana*  (Li et al. 2010) | *P. syringae* |  |  |  | *Auxin response factors* (*ARF*) | Increase PAMP |
| miR160 | *A. thaliana*  (Zhang et al. 2011b) | *P. syringae* |  |  |  | *ARF* | Increases PAMP |
| miR160 | *O. sativa*  (Li et al. 2014b) | *Magnaporthe oryzae* |  |  |  | *ARF* | Pathogen defence response |
| miR160 | *M. esculenta*  (Pinweha et al. 2015) | *Colletotrichum gloeosporioides* |  |  |  | *ARF* | Pathogen defence response |
| miR167 | 1. *thaliana*   (Fahlgren et al. 2007; Zhang et al. 2011b) | *P. syringae* |  |  |  | *ARF* | Pathogen defence response |

Table S2. (Continued)

| miR168 | *O. sativa*  (Wu et al. 2015) | *Rice Strip Virus*  (*RSV*) |  |  |  | *Argonaute 1* (*AGO1*) | Pathogen defence response |
| --- | --- | --- | --- | --- | --- | --- | --- |
| miR168 | *O. sativa*  (Wu et al. 2015) | *Rice dwarf virus* (*RDV*) |  |  |  | *AGO1* | Pathogen defence response |
| miR168 | *A. thaliana*  (Li et al. 2010) | *P. syringae* |  |  |  | *AGO1* | Pathogen defence response |
| miR319 | *O. sativa*  (Zhang et al. 2016c) | *Rice ragged stunt virus* *(RRSV)* |  |  |  | *Teosinte Branched1* (*TB1*) | Pathogen defence response |
| miR390 | 1. *thaliana*   (Zhang et al. 2011b) | *P. syringae* |  |  |  | *Trans-acting SiRNA3*  (*TAS3*) | Trigger the accumulation of ta-siRNAs during fungal attack |
| miR393 | *A. thaliana*  (Li et al. 2010) | *P. syringae* |  |  |  | *Transport inhibitor*  *response 1* (*TIR1*) | Pathogen defence response |
| miR398 | *A. thaliana*  (Li et al. 2010) | *P. syringae* |  |  |  | *Cu/Zn superoxide*  *dismutases* (*CSDs*)*, Copper Chaperone of CSD* (*CCD*) | Pathogen defence response |
| miR444 | *O. sativa*  (Wang et al. 2016c) | Rice strip virus (*RSV*) |  |  |  | *MADS* | Pathogen defence response |
| miR482 | *S. lycopersicum*  (Shivaprasad et al. 2012) | Turnip crinkle virus (*TCV*) |  |  |  | *Nucleotide-binding site leucine-rich repeat*  (*NBS-LRR*) | Induces R proteins |
| miR482 | *S. lycopersicum*  (Shivaprasad et., al., 2012) | *Cucumber mosaic virus* (*CMV*) |  |  |  | *NBS-LRR* | Pathogen defence response |

Table S2. (Continued)

| miR482 | *S. lycopersicum*  (Shivaprasad et al. 2012) | *Tobacco rattle virus* (*TRV*) |  |  |  | *NBS-LRR* | Pathogen defence response |
| --- | --- | --- | --- | --- | --- | --- | --- |
| miR482 | *S. lycopersicum*  (Ouyang et al. 2014) | *Fusarium oxysporum*  (*F. oxysporum)* |  |  |  | *NBS-LRR* | Pathogen defence response |
| miR528 | *O. sativa*  (Wu et al. 2017) | *Rice Strip Virus*  (*RSV*) |  |  |  | *Ascorbate oxidase* (*AO*) | Pathogen defence response |
| miR2118 | *C. arietinum*  (Kohli et al. 2014) | *F. oxysporum f.sp. ciceris* |  |  |  | *Toll/Interleukin-1 receptor-NBS-LRR*  (*TIR-NBS-LRR*) | Pathogen defence response |
| miR2118 | *S. lycopersicum*  (Shivaprasad et al. 2012) | *Cucumber mosaic virus* (*CMV*) |  |  |  | *NBS-LRR* | Induces the expression of R protein. |
| miR2118 | *S. lycopersicum*  (Shivaprasad et al. 2012) | *Tobacco rattle virus* (*TRV*) |  |  |  | *NBS-LRR* | Induces the expression of R protein. |
| miR2118 | *S. lycopersicum*  (Shivaprasad et al. 2012) | *Turnip crinkle virus* (*TCV*) |  |  |  | *NBS-LRR* | Induces the expression of R protein. |
| **Long ncRNAs** | | | | | | | |
| TalnRNA5 | *T. aestivum*  (Xin et al. 2011) | *Blumeria graminis f. sp. Tritici* (*B. graminis f. sp. Tritici*) |  |  |  | ND | Pathogen defence |
| TapmlnRNA8 | *T. aestivum*  (Xin et al. 2011) | *B. graminis f. sp. tritici* |  |  |  | ND | Pathogen defence |
| TalncRNA18 | *T. aestivum*  (Zhang et al. 2013b) | *P. striiformis f. sp. tritici* |  |  |  | ND | Pathogen defence |
| TapmlnRNA19 | *T. aestivum*  (Xin et al. 2011) | *B. graminis f. sp. tritici* |  |  |  | ND | Pathogen defence |

| TapmlnRNA30 | *T. aestivum*  (Xin et al. 2011) | *B. graminis f. sp. tritici* |  |  |  | ND | Pathogen defence |
| --- | --- | --- | --- | --- | --- | --- | --- |
| TalncRNA73 | *T. aestivum*  (Zhang et al. 2013b) | *P. striiformis f. sp. tritici* |  |  |  | ND | Pathogen defence |
| TalncRNA106 | *T. aestivum*  (Zhang et al. 2013b) | *P. striiformis f. sp. tritici* |  |  |  | ND | Pathogen defence |
| TalncRNA108 | *T. aestivum*  (Zhang et al. 2013b) | *P. striiformis f. sp. tritici* |  |  |  | ND | Pathogen defence |
| TAR-191 | 1. *thaliana*   (Zhu et al. 2014) | *F. oxysporum* |  |  |  | ND | Pathogen defence |
| TAR-197 | 1. *thaliana*   (Zhu et al. 2014) | *F. oxysporum* |  |  |  | *ND* | Pathogen defence |
| TAR-224 | *A. thaliana*  (Zhu et al. 2014) | *F. oxysporum* |  |  |  | *WRKY* | Pathogen defence |
| slylnc0195 | *S. lycopersicum*  (Wang et al. 2015c) | Tomato yellow leaf curl virus (*TYLCV*) |  |  |  | *Class III*  *homeodomain-leucine zipper* (*class III HD-Zip*) | Target mimic of miR166 |
| slylnc1077 | *S. lycopersicum*  (Wang et al. 2015c) | *TYLCV* |  |  |  | ND | Target mimic of miR399 |
| *ELF18-INDUCED lncRNA1* (*ELENA1*) | *A.thaliana*  (Seo et al. 2017) | *P.syringe* |  |  |  | *Pathogenesis-related gene1* (*PR1*) | Transcriptional regulation |
| LNC_001023 | *M.acuminata*  (Li et al. 2017) | *F. oxysporum* |  |  |  | *Pathogenesis-related protein* (*PRP*) *and Peroxidase*(*POX*) | Pathogen defence |

Table S2. (Continued)

| LNC_001474 | *M.acuminata*  (Li et al. 2017) | *F. oxysporum* |  |  |  | *PRP, POX* | Pathogen defence |
| --- | --- | --- | --- | --- | --- | --- | --- |
| LNC_002048 | *M.acuminata*  (Li et al. 2017) | *F. oxysporum* |  |  |  | *PRP, POX* | Pathogen defence |

**^a^Crop abbreviations:** ***P. trichocarpa****- Populus trichocarpa;* ***O. sativa****- Oryza sativa;* ***C. arietinum****- Cicer arietinum;* ***A. thaliana****-Arabidopsis thaliana;* ***S. lycopersicum****-* *Solanum lycopersicum;* ***T. aestivum****- Triticum aestivum;* ***M.acuminata****-* *Musa acuminata;* ***M. esculenta****- Manihot esculenta.*

**^b^Type of Pathogen: B**-Bacteria; **F**-Fungi; **V**- Virus.

**^c^Symbols:** represents down-regulation; represents up-regulation of small RNA species in the specified crop, **ND**- Not defined.
